# Effect of Carbon Nanofiber Clustering on the Micromechanical Properties of a Cement Paste

**DOI:** 10.3390/nano12020223

**Published:** 2022-01-10

**Authors:** Lesa Brown, Catherine S. Stephens, Paul G. Allison, Florence Sanchez

**Affiliations:** 1 Department of Civil and Environmental Engineering, Vanderbilt University, Nashville, TN 37212, USA; lesa.brown@vanderbilt.edu; 2 International Research Office, U.S. Army Engineer Research and Development Center, Ruislip HA4 7HB, UK; Catherine.s.stephens.civ@army.mil; 3 Department of Mechanical Engineering, The University of Alabama, Tuscaloosa, AL 35401, USA; pallison@eng.ua.edu

**Keywords:** carbon nanofibers, carbon nanotubes, micromechanics, elasticity, indentation, modulus, cement paste

## Abstract

The use of carbon nanofibers (CNFs) in cement systems has received significant interest over the last decade due to their nanoscale reinforcing potential. However, despite many reports on the formation of localized CNF clusters, their effect on the cement paste micromechanical properties and relation to the mechanical response at the macroscopic scale are still not fully understood. In this study, grid nanoindentation coupled with scanning electron microscopy and energy dispersive spectroscopy was used to determine the local elastic indentation modulus and hardness of a portland cement paste containing 0.2% CNFs with sub-micro and microscale CNF clusters. The presence of low stiffness and porous assemblage of phases (modulus of 15–25 GPa) was identified in the cement paste with CNFs and was attributed primarily to the interfacial zone surrounding the CNF clusters. The CNFs favored the formation of higher modulus C–S–H phases (>30 GPa) in the bulk paste at the expense of the lower stiffness C–S–H. Nanoindentation results combined with a microscale–macroscale upscaling homogenization method further revealed an elastic modulus of the CNF clusters in the range from 18 to 21 GPa, indicating that the CNF clusters acted as compliant inclusions relative to the cement paste.

## 1. Introduction

Carbon nanofibers (CNFs) are excellent candidates for reinforcement of cementitious materials at the nanoscale due to their unique characteristics (high aspect ratio with nanoscale diameters, and lengths of a few hundred nm to a few hundred µm; high strength; low density; and corrosion resistance) [1,2,3]. In addition to enhancing the mechanical properties and durability of cement-based materials, CNFs can perform new functions, such as stress-sensing, temperature monitoring, and electromagnetic shielding [4,5,6,7,8,9,10,11,12]. Potential applications of CNFs in cement-based materials include infrastructure health monitoring, traffic monitoring, and where low density-high performance materials are important. While there have been reports that the addition of CNFs can provide sub-micron crack bridging [13,14,15,16] and increase the amount of high-density C–S–H [17], the full reinforcing potential of the CNFs has largely been hindered by difficulties dispersing the CNFs in the cement matrix. Agglomerations of CNFs have been shown to form as localized sub-micro- and microscale clusters within the cement matrix due to the ubiquitous van der Waals forces and the unique environment of the hydrating cement paste (high pH, ionic composition, and cement grain size) [13,15,18,19,20,21]. While the occurrence of CNF clustering may often be viewed as a flaw or imperfection (i.e., randomly oriented, individually embedded CNFs with a uniform spatial arrangement would typically provide better load transfer than clustered CNFs), it may be beneficial in some situations. For example, clustering has been shown for polymer composites to enhance certain mechanical properties, including fracture toughness and damage tolerance [22], and to improve the formation of a percolating network for electrical conductivity [5,23,24]. Similar results for cementitious composites have been reported with a lower electrical resistance change due to micro-cracking observed in the presence of agglomerated carbon nanotubes (CNTs) compared to well-dispersed CNTs, suggesting that controlled agglomeration of CNFs/CNTs may be desirable depending on the application [5]. CNF clusters have also been found to create localized porosity [25] while providing a reinforcing network that has been reported by the authors to influence the macroscale flexural response [13] and delay cracking and spalling during sulfate-induced degradation [14]. Many studies have focused on the macroscale mechanical, physical, and electrical properties of CNT/CNF-modified cement pastes [4,6,7,9,10,22,26,27,28,29,30,31,32,33,34,35,36,37,38,39,40,41,42,43,44,45,46], but few studies have related the macroscale response to the dispersion state(s) of the CNFs [8,13,15,20,47,48,49,50]. As a result, the local influence of the CNFs, CNF clusters, and their interface with the surrounding matrix on the microstructure and mechanical behavior of the cement paste is not well understood. Given that CNF clusters might be inevitable in the cement matrix, possibly desirable for some applications, and their expected role in the mechanical properties and performance of CNF-modified cement pastes, the goal of this study was to evaluate the effect of CNF clustering on the cement paste micromechanical properties in order to better understand the structure–property relationships between the CNFs, CNF clusters, and the cement paste. Understanding these relationships provides direction for developing strategies in tuning the mechanical properties of the composite. Additionally, knowledge of the micromechanical properties of CNF clusters can provide insight into their stability under mechanical stress and thus inform mechanical dispersion methods.

The specific objectives of this paper were to (i) characterize the effect of CNFs and CNF clusters on the local micromechanical properties of a cement paste, (ii) correlate the micromechanical properties to microstructural and chemical properties, and (iii) link the microscale behavior with the overall mechanical response of a CNF-modified cement paste. Grid nanoindentation coupled with scanning electron microscopy (SEM)/energy dispersive spectroscopy (EDS) was used to determine the local elastic indentation modulus and hardness of a cement paste containing 0.2% CNFs with CNF clusters. Grid nanoindentation was chosen for this study as it provided a means of characterizing both the bulk cement paste and the areas surrounding the CNF clusters while avoiding a bias in the selection of the indent locations, which is important in the presence of a heterogeneous distribution of phases and inclusions at various length scales. Grid nanoindentation relies on a large number of indents and statistical deconvolution of experimental data to extract the mechanical properties of mechanically distinct phases and has been shown in the literature to be the method of choice for heterogeneous materials, such as cement pastes [51,52,53,54,55,56,57]. In addition, statistical analysis of the results provides information regarding the distribution and volume fraction of the mechanical material phases derived from the deconvolution process that are then used as input information for upscaling the micromechanical properties to the macroscale. A statistical Gaussian fitting method was used to segment the nanoindentation data into mechanically distinct phases. Grid nanoindentation was supplemented by backscattered electron (BSE) image analysis and energy dispersive spectroscopy (EDS) analysis at each nanoindentation location to relate the mechanical phases identified statistically by Gaussian deconvolution to the chemical phases present in the microstructure and to thus provide quality control of the data set. In addition, to further identify the nanoscale reinforcing behavior of the CNF clusters and their influence on the macroscale, a microscale–macroscale analytical upscaling approach described in [58] was applied to the nanoindentation dataset of the cement paste with CNFs. This approach has been shown, for an ordinary portland cement paste, to effectively upscale nanoindentation data at the microscale to the macroscale for comparison with macroscale mechanical measurement of the elastic modulus obtained via a pulse velocity method [58]. The paper provides new insights into the property–microstructure relations for CNF-modified cement pastes, particularly the effect of CNF agglomeration, and, to the best of the authors’ knowledge, reports for the first time, mechanical measurements of the interfacial zone between the CNF clusters and the cement matrix and estimated values of the elastic modulus of the CNF clusters. Significantly, the research demonstrated that grid nanoindentation with constitutive phase analysis combined with a micro–macroscale upscaling method is a valuable approach to estimate properties of inclusions that cannot be directly measured experimentally and to link the local micromechanical properties of a multiphase material that has randomly distributed inclusions at various length scales to its macroscopic mechanical response.

## 2. Materials and Methods

### 2.1. Cement Paste Preparation

Two cement pastes were prepared: a plain cement paste (reference cement paste) and a cement paste containing 0.2% CNFs per mass of cement (cement paste with CNFs). A dosage of 0.2% CNFs was selected for this study as it is in the median range of typical dosages found in the literature and was expected to demonstrate a range of CNF dispersion (i.e., individual fibers to microscale clusters). Type I/II Portland cement (Lafarge, Nashville, TN, USA) and Pyrograf^®^ III PR 19 LHT CNFs (Applied Sciences, Inc., Cedarville, OH, USA) were used. Glenium^®^ 7500 (BASF, Ludwigshafen, Germany), a polycarboxylate-based high range water reducer (HRWR), was used at a loading of 1% per mass of cement to promote the dispersion of the CNFs in the cement paste [20,26,59]. A water to cement (w/c) ratio of 0.28 was used for all mixes. The HRWR, CNFs (where applicable), and water were combined and sonicated with a bath sonicator (Aquasonic model 250D, VWR International, Radnor, PA, USA) for 30 min prior to mixing with the cement powder. After mixing, the paste was poured into 2.54 cm × 2.54 cm × 69 cm (H × W × L) beam molds and compacted by hand. The beams were de-molded after 24 h and kept at room temperature under 100% relative humidity for four years before testing.

### 2.2. Microscale Analysis

#### 2.2.1. Grid Nanoindentation

**Figure 1:**
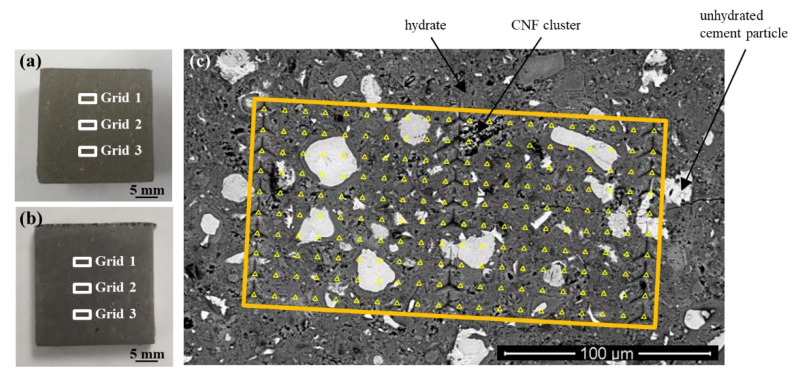
Cross-section of the (**a**) reference cement paste and (**b**) cement paste with CNFs showing the location of the nanoindentation grids and (**c**) SEM image of a representative nanoindentation grid showing 200 indents (yellow triangles are enlarged to show indent locations).

Cement paste beams were cross-sectioned using a low-speed diamond saw, epoxy-mounted, and polished. Details of the mounting and polishing procedure can be found in [58]. The polishing procedure resulted in an average sample surface roughness (R_a_) of 40–60 nm for a scan size of 25 µm × 25 µm, as measured by scanning probe microscopy. This average sample surface roughness has been reported in the literature to be adequate for the indentation of cement phases with a penetration depth of 200–300 nm [51,56,60,61,62,63,64,65,66]. A diamond Berkovich tip and an Agilent Nanoindenter G200 Testing System (Agilent Technologies, Santa Clara, CA, USA) were used to perform nanoindentation of the reference cement paste and cement paste with CNFs. A fused silica standard with known mechanical properties and a second-order area function was used to calibrate the tip [67]. Nanoindentation was performed using a grid collecting technique in which a total of 600 indents (three grids of 200 indents) were collected on each cement paste (Figure 1). Indent spacing was 10 µm in both the X and Y directions. A spacing of 10 µm between indents has been shown to be sufficient in cementitious materials for preventing adjacent indents from influencing the next indentation result [51,52,56,61,66,68,69,70]. A maximum force of 2 mN was applied during a 10 s loading period, with a targeted strain rate of 0.050 s^−1^. A maximum load of 2 mN was selected as it targeted indentation depths ranging from 200 to 300 nm, which, according to the literature, allows the individual response of C–S–H to be captured while also overcoming the surface roughness of polished cementitious materials [71,72]. The maximum load was held for 15 s and followed by a 10 s unloading period. The beginning, middle, and end of each grid were marked with fiducial indents to provide for clear identification of the grid during SEM/EDS analysis. Each indentation curve was then evaluated individually, and abnormal curves due to cracking of the immediate surrounding sample area during testing or improper contact between the tip and sample were discarded so as not to interfere with the determination of the micromechanical properties (Figure S1). Curve abnormality was generally seen for the large CNF clusters that, by their nature (i.e., composed of an entangled mass of fibers versus a hard solid surface), violated the assumption of a hard, flat surface needed for their indentation. The invalid load–displacement curves from the CNF clusters were properly removed from the statistical analysis per the accepted data evaluation for nanoindentation and were, therefore, not included in the determination of the micromechanical properties and were, instead, determined from a modeling procedure. Even after abnormal curve removal, over 400 valid indents were characterized for each cement paste. This number of indents has been reported to be sufficient for differentiating between primary cementitious phases (e.g., C–S–H, CH, or unhydrated cement particles) [66,71,73,74,75]. The indentation modulus and hardness of each valid indent were calculated using the Oliver and Pharr method [76], where Poisson’s ratio was assumed to be 0.3 for all calculations [77,78].

#### 2.2.2. Gaussian Fitting Procedure

Each indentation modulus and hardness nanoindentation dataset was segmented into mechanically distinct phases using the statistical multimodal Gaussian fitting procedure used in [58]. The experimental probability density function (PDF) was plotted and fit with n Gaussian distributions. The parameters (i.e., mean, µ, and standard deviation, σ) for each fitted Gaussian distribution were then calculated, and the theoretical PDF based on the fitted Gaussian distributions was generated. The number of characteristic peaks in the experimental PDF was used to initially set the starting number of Gaussian distributions, n. A looping algorithm that allowed for multiple trials was coupled with an expectation maximization (EM) algorithm to determine different possible theoretical PDFs and to iteratively test for the appropriate number of Gaussian distributions. Only Gaussian distributions corresponding to the primary peaks of the experimental PDF (i.e., peaks composed of a minimum of 5% of the total indents) were considered for further analysis. A bin size of 1 GPa was used for all the Gaussian indentation modulus fits, which has been shown to be appropriate for cement pastes [56,79,80]. This bin size provided the best approximation of the raw data based on multiple bin sizes (0.1 to 10 GPa) examined (Figure S2). A discussion on the effect of the bin size can be found in [81,82]. A bin size of 0.1 GPa was used for all of the hardness fits [56]. The overall best fit (i.e., theoretical PDF) from all the trials was selected based on comparison to the experimental PDF by the Kullback–Leibler divergence [83,84]. As an additional verification of the final fit and bin size selection per [81], the theoretical and experimental cumulative density functions (CDFs) were also plotted and compared and are provided in Supplementary Materials (Figures S3 and S4).

#### 2.2.3. Chemical and Microstructural Characterization

A coupled SEM/EDS analysis was used to identify the phase(s) present at each indent location—hydrates, unhydrated cement particles, flaws, or a combination of these. An environmental FEI Quanta FEG 650 high-resolution scanning electron microscope (FEI company, Hillsboro, OR, USA) equipped with a Schottky field emission gun, digital imaging, and an energy dispersive X-ray spectrometer was used to collect secondary (SE) and backscatter electron (BSE) images of the nanoindentation grid locations and chemical data at the location of each indent. A pressure of 130 Pa (in ESEM mode), accelerating voltage of 15 kV, spot size of 3.5, and a working distance of 10.5 mm were used.

### 2.3. Macroscale Analysis

#### 2.3.1. Pulse Velocity Measurements of Elastic Modulus

The pulse velocity of the cement pastes was measured using a Pundit PL 200 with 150 kHz transducers (Proceq, Schwerzenbach, Zürich, Switzerland). Pulse velocity measurements are non-destructive tests that yield results that closely mimic the inherent Young’s modulus of the composite. The dynamic modulus of elasticity was calculated according to ASTM C597: Standard Test Method for Pulse Velocity through Concrete [85].

#### 2.3.2. Microscale–Macroscale Analytical Homogenization

A homogenization approach based on the analytical Mori–Tanaka method [51,86] was employed to upscale the micromechanical properties obtained from grid nanoindentation to the macroscale and estimate the elastic modulus of the CNF clusters. The methodology used in the homogenization approach has been detailed in [58]. The mechanically distinct phases identified during the Gaussian fitting of the nanoindentation datasets were used to define the reference matrix and inclusions to be used in the Mori–Tanaka method. The volume fraction associated with porosity was estimated using SEM porosity analysis and then corrected for the fraction of porosity outside the SEM domain as described in [58]. The volume fractions of each mechanically distinct phase were determined from the results of the Gaussian fitting procedure and adjusted to account for the porosity. For the cement paste with CNFs, an additional phase was defined to account for the nanoscale reinforcing behavior of the CNF clusters (cluster pull-out, cluster crack deflection, etc.) [13]. This behavior could not be captured directly from experimental nanoindentation because of the nature of the CNF clusters—the random packing of the CNFs does not yield a hard, flat, stable surface that would be appropriate for surface indentation. A multi-step process was, therefore, employed to develop an acceptable cluster phase to characterize the CNF cluster micromechanical behavior in the homogenization approach. This process involved varying Poisson’s ratio of the CNF cluster phase to back-calculate the elastic modulus of the cluster phase in order to approximate an overall homogenized elastic modulus that corresponded to the experimentally measured elastic modulus of the cement paste with CNFs.

## 3. Results and Discussion

### 3.1. CNF Clusters and Cluster Interfacial Zone

**Figure 2:**
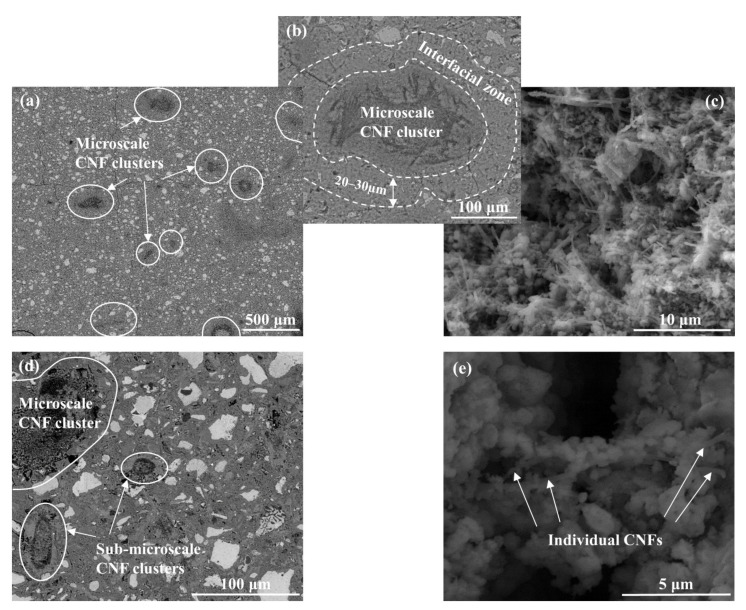
SEM images showing (**a**) the distribution of microscale CNF clusters in cement paste, (**b**) an individual CNF cluster and surrounding interfacial zone, (**c**) the entangled mass of CNFs and cement hydrates within a cluster, (**d**) the distribution of sub-microscale CNF clusters in cement paste, and (**e**) individual CNFs that were well embedded in the cement paste.

Both individually dispersed and clustered CNFs were seen throughout the hydrated cement paste. The individually dispersed CNFs were well embedded in cement hydrates and in close proximity to only a few other fibers (Figure 2e). The clustering of CNFs formed both microscale (clusters with a diameter above 100 µm that could be easily identified via optical microscopy; Figure 2a) and sub-microscale (clusters with a diameter below 100 µm that needed to be identified via SEM; Figure 2d) CNF clusters. The clusters were entangled masses of CNFs with inter-fiber pore networks (Figure 2b,c) that induced localized zones of higher porosity and were scattered throughout the cement paste cross-section [14,25]. Cement hydrates were also found scattered within the clusters (Figure 2c), and previous work by the authors identified the presence of CH plates within the clusters [25]. Approximately 40–60% (Assumes a CNF density of 0.032 g/cm^3^ per the manufacturer’ specifications [87], cluster shapes that range from spherical to ellipsoidal, and a characteristic length of 0.27 mm (based on the average Feret diameter of the microscale clusters) in order to transform from surface area to volume.) of the total CNFs added during mixing (i.e., 0.2% per mass of cement × 40 to 60%) were thought to be present as microscale clusters in the final hydrated cement paste; Figure 2a. The microscale clusters ranged in size from 125 to 1500 µm and had an average Feret diameter of 270 µm, indicating that the clusters were more concentrated towards the lower end of the size distribution. The microscale clusters had an average aspect ratio of 2.0 ± 0.96 and an average circularity of 0.69 ± 0.18, suggesting that the clusters had a more ellipsoidal shape (Figure 2b) compared to the spherical shape typically associated with air voids in cement paste. Systematic measurements of the sub-microscale CNF clusters were more difficult due to their low visibility on a polished surface and were primarily accomplished via SEM imaging. The sub-microscale CNF clusters ranged in size from just a few microns up to ca. 50 µm in diameter. A similar ellipsoidal shape was also seen for the sub-microscale clusters as well as the presence of cement hydrates inside the cluster.

Previous work by the authors [25] indicated that each CNF cluster was surrounded by an interfacial zone, which had distinctive microstructural and chemical signatures compared to the surrounding cement paste. BSE imaging revealed that this interfacial zone was characterized by a greater porosity (ca. 34% increase in porosity as measured by an SEM porosity counting technique) compared to the surrounding cement paste [25] and by the absence of unhydrated cement particles (Figure 2b). EDS mapping of the sample surface also indicated a lower calcium content and higher potassium content in the interfacial zone compared to the surrounding cement paste [25]. The interfacial zone was 20–30 µm thick, with size generally independent of the size of the CNF cluster, and served as a bridge between the cement matrix and the clusters (Figure 2b).

### 3.2. Local Indentation Modulus and Hardness

**Figure 3:**
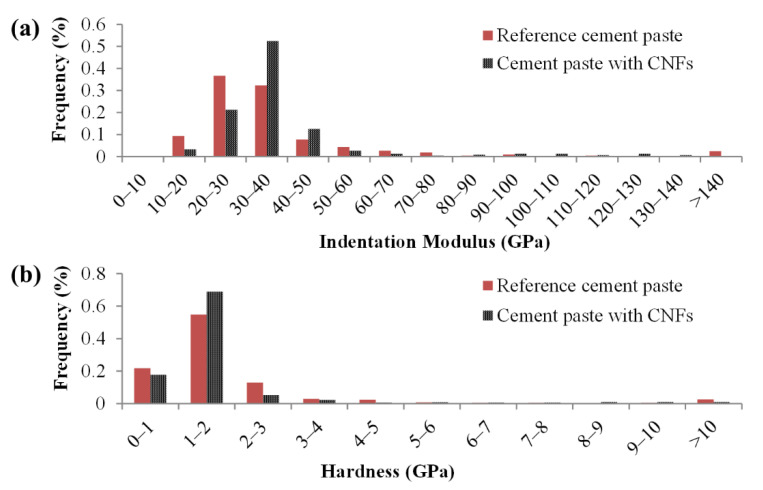
Histograms of the indentation (**a**) modulus and (**b**) hardness for the reference cement paste (414 valid indents) and the cement paste with CNFs (479 valid indents).

**Figure 4:**
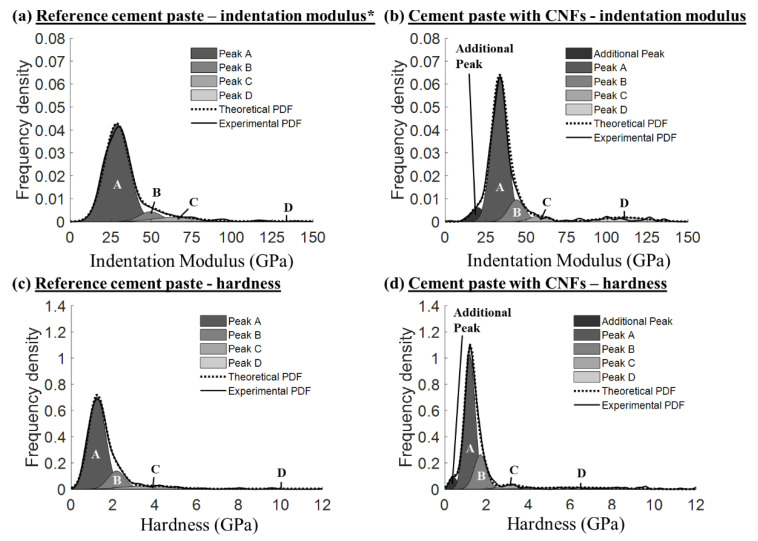
Multimodal Gaussian fitting results for the indentation modulus (**a**,**b**) and hardness (**c**,**d**) of the reference cement paste and cement paste with CNFs, respectively. * Indentation modulus data of the reference cement paste have been reported in [58].

The effect of the CNFs on the cement paste micromechanical properties, as evidenced by an increase in the number of indents with indentation modulus values between 30 and 50 GPa (Figure 3a) and number of indents with hardness values in the 1 to 2 GPa range (Figure 3b), suggested that the CNFs influenced the stiffness and hardness of the primary hydrates (e.g., C–S–H phases). The combination of both well-dispersed CNFs and CNF clusters found in the composite were thought to influence the stiffness and hardness of the hydrates, primarily through a reinforcement effect at the nanoscale for the well-dispersed CNFs and a physical filling effect of the CNF clusters which led to an improved packing density and lower porosity of the surrounding bulk cement paste [25]. A similar effect has been reported with the incorporation of carbon nanotubes in cement pastes [48]. Gaussian fitting of the nanoindentation data revealed the presence of four (4) primary peaks for the reference cement paste and four (4) corresponding primary peaks for the cement paste with CNFs (Figure 4) plus an additional primary peak that was centered around low indentation modulus and hardness values (18–23 GPa and 0.2–0.7 GPa, respectively).

Normalized histograms of the raw data can be found in Figure S2, and a comparison of the theoretical and experimental CDFs can be found in Figure S3. The four primary peaks seen in both cement pastes corresponded to mechanically distinct peaks typically discussed in the literature for cement-based materials and were, for both cement pastes, labeled as Peaks A, B, C, and D (for indentation modulus values, Peak A is typically centered around 20–35 GPa, Peak B around 40–50 GPa, Peak C around 50–65 GPa, and Peak D around 100–150 GPa) [51,56,72]. The additional peak identified in the cement paste with CNFs encompassed ca. 10% of the total valid indents and was indicative of the presence of a porous assemblage of hydrated phases that exhibited an overall low stiffness. Visual examination by SEM of the location of the indents corresponding to this additional peak indicated that the majority of the indents were in or near CNF clusters or present in areas that may have included sub-micro-CNF clusters (i.e., area of higher porosity). The interfacial zone surrounding the CNF clusters was thought to have contributed to this additional peak during nanoindentation. A difference in the position of the four (4) primary peaks (Peaks A, B, C, and D) was also observed between the two cement pastes, with a statistically higher indentation modulus value for Peak A with CNF addition and an overall shift toward lower indentation modulus and hardness values for Peaks B, C, and D (Table 1; Welch’s *t*-test, α = 0.05). While Peak A was the predominant peak in both cement pastes, the cement paste with CNFs had a lower percentage of indents associated with Peak A (63% vs. 78%) and a higher percentage of indents associated with Peak B (16% vs. 11%), indicating a shift towards phases of higher indentation modulus values in the presence of CNFs.

**Figure 5:**
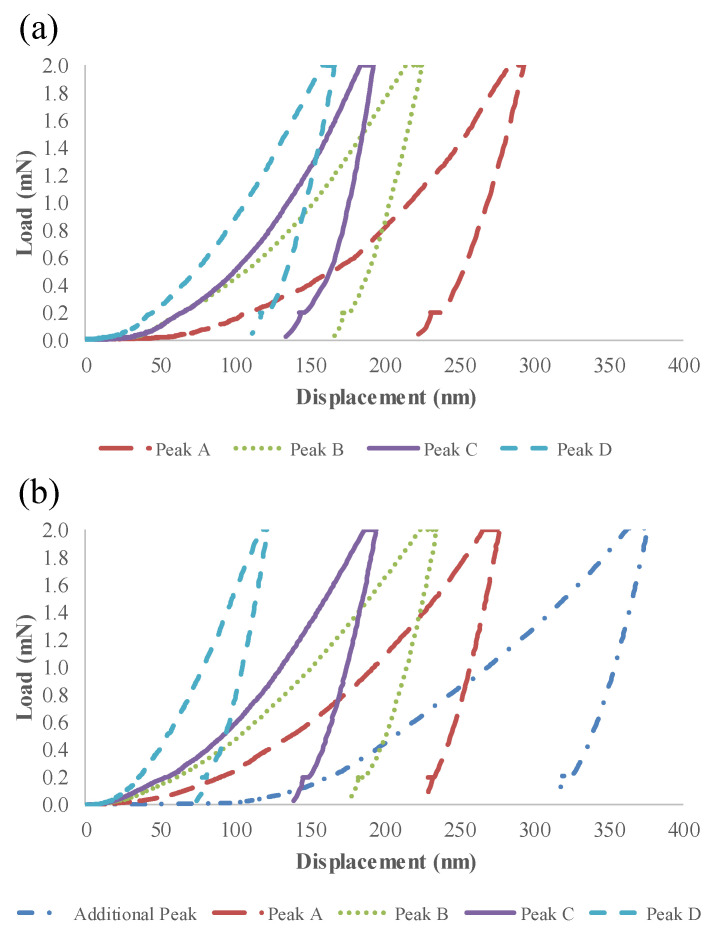
Load displacement curves for the mechanically distinct peaks identified in (**a**) the reference cement paste and (**b**) the cement paste with CNFs.

In agreement with the hardness values, the average maximum displacement of the indent load–displacement curves decreased as the hardness increased from Peak A to Peak D (Figure 5). The indents associated with the cluster interfacial zones demonstrated a higher average maximum displacement (390 nm) than that of all the other indents from both cement pastes. In addition, lower average maximum displacements were seen for the indents associated with Peak A of the cement paste with CNFs compared to that of the reference cement paste (276 nm vs. 292 nm, respectively) and Peak D of the cement paste with CNFs (121 nm vs. 154 nm, respectively). In contrast, the indents associated with Peaks B and C had similar average maximum displacements for both cement pastes (234 nm vs. 225 nm and 195 nm vs. 193 nm, respectively). The higher indentation modulus value, lower percentage of indents, and lower average maximum displacement for the indents associated with Peak A for the cement paste with CNFs clearly indicated that the CNFs influenced the phases associated with this dominant Peak A.

### 3.3. Coupled Grid Nanoindentation with SEM Analysis

**Figure 6:**
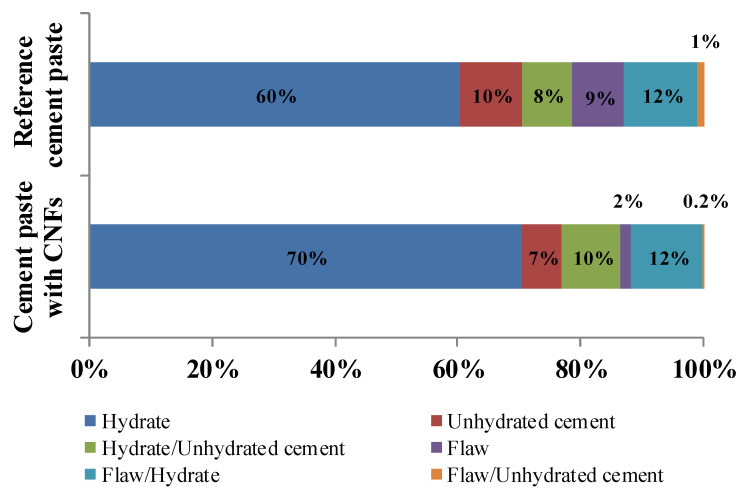
Visual microstructural classification of indents based on SEM image analysis for the reference cement paste and the cement paste with CNFs.

**Figure 7:**
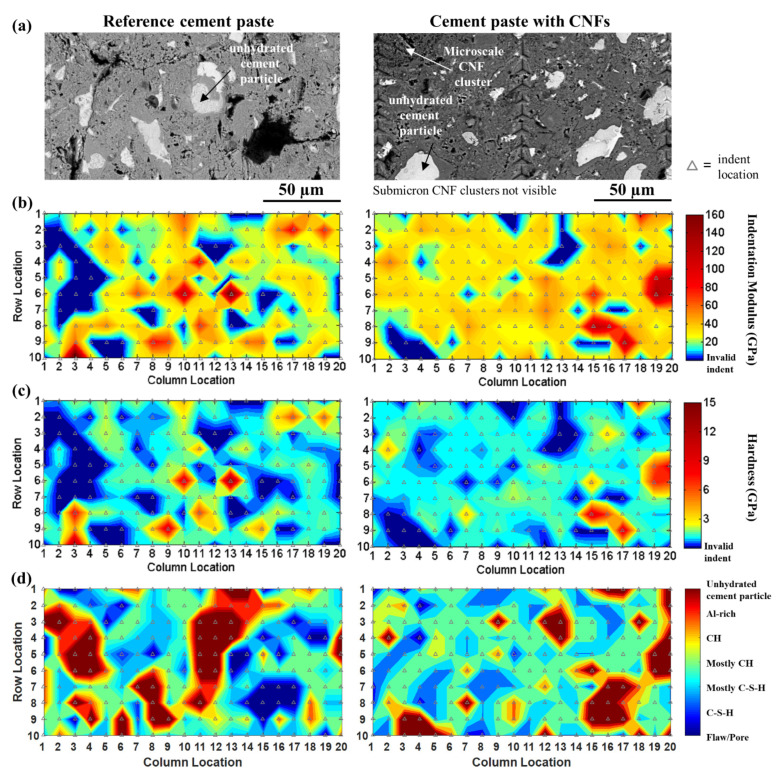
(**a**) SEM backscattered electron (BSE) image of a representative nanoindentation grid and corresponding (**b**) indentation modulus, (**c**) hardness, and (**d**) chemical phase contour maps for the reference cement paste (**left**) and the cement paste with CNFs (**right**).

Classification of the nanoindentation indents based on their visual microstructural attributes as identified using SEM (Figure 6) indicated that the cement paste with CNFs had a lower number of flaws/porosity (1.8% vs. 8.5%) compared to the reference cement paste. The difference in flaws/porosity was consistent with the overall more uniform distribution of indentation modulus and hardness seen in the contour maps of the cement paste with CNFs (Figure 7) and the results found in [25], which showed that the presence of CNFs provided for a pore refinement of the cement paste. A higher hydrate content (70.3% vs. 60.3%) with a lower unhydrated cement particle content (6.7% vs. 10.0%) was also found for the cement paste with CNFs, suggesting a higher degree of hydration in the presence of CNFs. A similar influence on the degree of hydration has been reported in the literature for carbon nanotubes (CNTs) [46,88], where the CNTs were thought to provide a filling effect in the pore network that allowed for better bonding and bridging of cement phases during hydration and may have facilitated the accelerated growth of some hydration products. Furthermore, the preferential formation of CH plates within the CNF clusters during hydration and the relative hydrophobicity of the CNF clusters was thought to influence the resulting calcium hydrate phases of the surrounding cement paste and may have helped promote the distribution of C–S–H.

The mechanically identified peaks were correlated to chemical phases based on indentation modulus and hardness measurements of cement phases reported in the literature from nanoindentation (Table 2; [52,53,56,60,61,63,64,66,71,72,80,89,90,91,92,93,94,95,96]): (i) Additional Peak—porous assemblage of main hydration products; (ii) Peak A—main hydration products; (iii) Peak B—high stiffness hydration products; (iv) Peak C—CH and unhydrated cement particles (or a combination of the two); and (v) Peak D—unhydrated cement particles. The higher mean indentation modulus value of Peak A (main hydration products, such as C–S–H) and a higher percentage of Peak B indents (thought to be CH or high stiffness C–S–H) seen in the Gaussian fitting of the indentation modulus values suggested that the CNFs influenced the formation of the main hydration products of the cement paste and played a role in the formation of higher stiffness hydration products.

### 3.4. Hydrate Phase Segmentation by EDS Analysis

**Figure 8:**
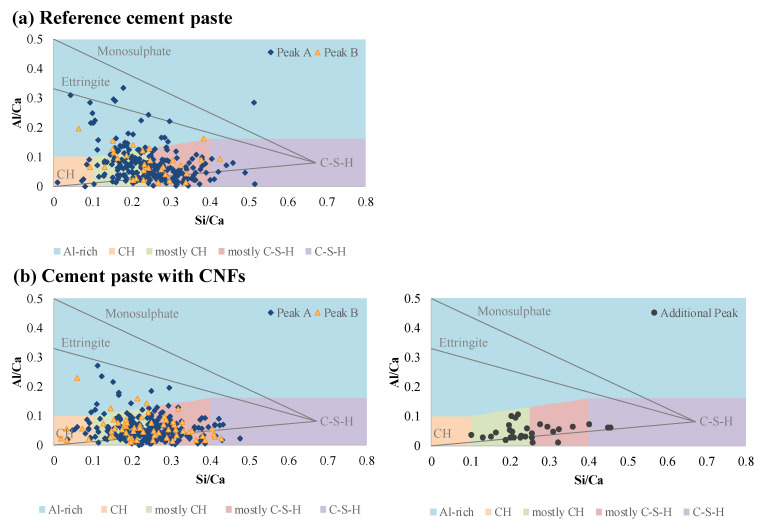
Segmentation of hydrates by chemical composition using SEM/EDS analysis for the (**a**) reference cement paste and (**b**) cement paste with CNFs.

The chemical phase contour maps (Figure 7d) indicated a shift towards higher stiffness hydration products for the cement paste with CNFs as well as a decrease in the unhydrated cement particles. For both cement pastes, the Al-rich phases were found to be in close proximity to the unhydrated cement particles, followed by higher stiffness hydration products, such as CH, and main hydration products, such as C–S–H distributed in between. Plots of atomic ratios of aluminum to calcium (Al/Ca) versus silicon to calcium (Si/Ca) obtained from EDS analysis of the indents identified as hydrates showed that the majority of the hydrates of both cement pastes (ca. 77%) corresponding to mechanically distinct Peaks A and B were distributed along the CH/C–S–H line and represented a mixture of CH and C–S–H phases that were associated predominantly with Si/Ca ratios ranging from 0.1 to 0.4 (Figure 8). These results indicated that indentation likely occurred on a mixture of phases rather than pure phases and reflected the highly heterogeneous nature of hydrated cement pastes. A similar result has been reported in the literature [72,81]. EDS analysis further revealed that the cluster interfacial zones associated with the Additional Peak were primarily composed of mostly CH and C–S–H phases and did not contain ettringite or monosulfate phases, unlike the bulk of the cement matrix (Figure 8). This lack of Al-rich phases was consistent with the lack of unhydrated cement particles in the interfacial zone of the CNF clusters and the preferential proximity of the Al-rich phases to the unhydrated cement particles in the bulk cement matrix as seen from the spatial chemical phase contour maps (Figure 7d). The composition of the cluster interfacial zones was similar to that typically found at interfacial transition zones between cement paste and aggregates and was consistent with the Le Chatelier hydration process which favored the preferential formation of CH and C–S–H in and around the CNF clusters [102,103].

**Figure 9:**
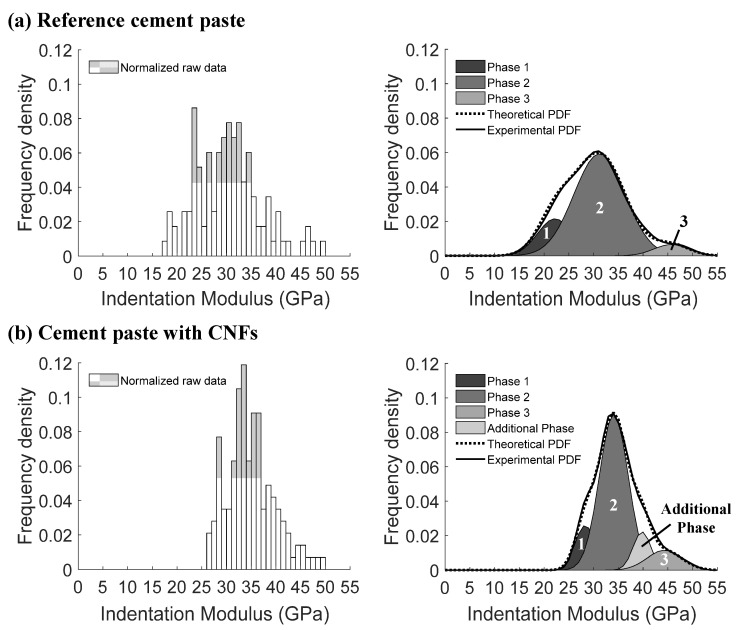
Normalized histogram of the raw data (**left**) and multimodal Gaussian fitting results (**right**) for the 0 to 50 GPa range of the indentation modulus typically associated with C–S–H phases for the (**a**) reference cement paste and (**b**) cement paste with CNFs.

Statistical deconvolution of the PDF Gaussian fit of the indentation modulus data for the indents corresponding to the hydrates that were within the 0 to 50 GPa range and demonstrated an EDS chemical signature that was indicative of a C–S–H phase indicated the presence of three (3) phases for the reference cement paste and four (4) phases for the cement paste with CNFs (Figure 9, comparison of the empirical and theoretical CDF can be seen in Figure S4). The cluster interfacial zones identified in the cement paste with CNFs (porous assemblages of CH and C–S–H phases) were not considered in this analysis. Based on indentation modulus values provided in the literature for the 0 to 50 GPa range typically associated with C–S–H phases, Phase 1 of the reference cement paste likely corresponded to low stiffness C–S–H (indentation modulus values below 30 GPa) [52,53,60,71,91,92,98], Phase 2 to high stiffness C–S–H (indentation modulus values between 30 and 35 GPa) [52,53,60,71,91,92,98], and Phase 3 to ultra-high stiffness C–S–H (indentation modulus values above 40 GPa) [104] (Table 3). The PDF deconvolution showed that the high stiffness C–S–H dominated over the low stiffness C–S–H in the nanomechanical response of the reference cement paste, while the ultra-high stiffness C–S–H represented only a minor volume proportion. These results were consistent with the literature, which showed that for cement pastes with low w/c ratios (<0.35), a higher ratio of high-density C–S–H vs. low-density C–S–H was expected [104,105,106,107]. For the cement paste with CNFs, a statistically higher indentation modulus value for Phase 1 than that of the reference cement paste was observed, indicating a shift of the indentation modulus of the low stiffness C–S–H phase with the addition of the CNFs towards the high end of the range of values typically found in the literature for low stiffness C–S–H [52,53,60,71,91,92,98]. In addition, the volume proportion of the low stiffness C–S–H phase decreased in favor of the higher stiffness C–S–H phases (Table 3). A similar result has been reported in the literature for CNFs [108] as well as the addition of CNTs, where the CNTs were reported to increase the formation of high-density C–S–H over that of low-density C–S–H [48,95,96]. This shift in the C–S–H phase distribution and properties occurring for the cement paste with CNFs was thought to be due to the relative hydrophobicity of the CNF clusters, providing for better hydration external to the clusters, and a filling effect of the clusters that favored a higher packing density of the surrounding cement matrix. Well-dispersed CNFs were also thought to play a role in the shift towards higher stiffness C–S–H by providing a reinforcing effect in the nanoporosity of the phase. A similar effect on C–S–H has been reported in the literature for both CNFs [108] and CNTs [48]. An intermediate phase of statistically significant volume proportion was furthermore identified between the high stiffness and ultra-high stiffness C–S–H phases in the cement paste with CNFs. This phase was thought to correspond to a mixture of CH and C–S–H phases that yielded indentation modulus values that exceeded high stiffness C–S–H. The evidence of C–S–H/CH nanocomposites has been previously reported in the literature [72]. It was also thought that the shift towards higher stiffness C–S–H phases with the addition of CNFs was influenced by the preferential formation of CH within the CNF clusters (reported in [25]), possibly at the expense of the lower stiffness C–S–H.

### 3.5. Estimation of the Elastic Modulus of the CNF Clusters through Nanoindentation-Based Microscale–Macroscale Upscaling Approach

A nanoindentation-based microscale–macroscale upscaling method was used to estimate the elastic modulus of the CNF clusters because direct indentation on the surface of the CNF clusters was not possible as the entangled fibers did not provide a hard, flat surface that would be appropriate for nanoindentation. Yet, CNFs located near the edge of the clusters appeared to be embedded in the interfacial zone and cement hydrates (primarily CH plates) were found scattered within the CNF clusters [14,25], suggesting that the CNF clusters likely have the capacity for load transfer and to influence cracking behavior, which in turn, can impact the macroscale mechanical properties of the material. An estimate of the elastic modulus of the CNF clusters was therefore of interest to better understand the influence of the CNF clusters and interfacial zones on the macroscale properties of the composite.

The elastic modulus of the CNF clusters was determined by using a microscale–macroscale upscaling homogenization method that was informed by the local micromechanical moduli obtained from grid nanoindentation results to account for the effective, mechanical matrix phases and by the macroscale measurement of the elastic modulus obtained by pulse velocity. The mechanically identified Peak A (main hydrates) was considered the reference matrix phase for the Mori–Tanaka portion of the upscaling method, while the mechanically identified peaks B through D, the cluster interfacial zone, porosity, and the cluster phase were considered inclusion phases. The only independent/unknown variable of the microscale–macroscale upscaling homogenization method was the modulus of the “cluster phase”, which due to the nature of the model equations, was solved by using a standard, implicit, iterative procedure. All of the other parameters of the model were either characterized experimentally or were calculated based on experimental data.

Measurement of the pulse velocity of the cement paste with CNFs provided an experimental dynamic elastic modulus of 29.5 GPa. The volume fraction of the cluster phase was estimated to be 5% based on an analysis of CNF cluster surface area coverage (Assumes a cluster volume based on the measured Feret diameter of each cluster and cluster shapes that range from spherical to ellipsoidal; a characteristic length of 0.27 mm (based on the average Feret diameter of the clusters) was used to transform from surface area to volume. The size of the microscale clusters controlled the volume fraction of the cluster phase.) and Poisson’s ratio of the clusters was varied from 0.1 to 0.4 to cover the range exhibited by most materials and account for the fact that the CNF clusters were complex and random networks of entangled, flexible fibers and high porosity that deform differently depending on their packing density. The determination of the elastic modulus of the cluster phase was not sensitive to Poisson’s ratio and revealed elastic modulus values that ranged from 18 to 21 GPa (Table 4). The elastic modulus of the cluster phase was determined to be similar in magnitude to that of the cluster interfacial zone and had a non-negligible contribution to the homogenized elastic modulus (31.7 GPa without including the elastic modulus of the cluster phase vs. 29.5 GPa with the cluster phase). While the elastic modulus value of the CNF clusters is based on an estimate of the volume fraction of the CNF clusters, including the cluster phase provided evidence that the CNF clusters themselves imparted nanoscale reinforcement to the cement paste.

The CNF clusters and cluster interfacial zones exhibited the lowest stiffness of all the primary matrix phases, and the clusters acted as small, compliant inclusions relative to the matrix. This result was consistent with the improvement in the flexural toughness reported in the presence of CNF clusters by the authors [13,25]. The edges of the CNF clusters were thought to have embedded fibers that provided nanoscale reinforcement at the interfacial region and influenced the overall cluster mechanical behavior. The role of compliant inclusions relative to the matrix in matrix toughening mechanisms has been described in the literature [109,110,111]. The elastic moduli of the main hydration products (C–S–H phases, Phase A), cluster phase, and cluster interfacial zone controlled the macroscale elastic modulus of the cement paste with CNFs, indicating that the CNFs and CNF clusters played a significant role in the macroscopic response of the material and in improving flexural toughness.

## 4. Conclusions

The micromechanical properties of cement pastes with and without CNFs were studied using grid nanoindentation and correlated with microstructural observations and chemical analysis at each indentation site. The addition of CNFs clearly influenced the micromechanical properties of the cement paste. The presence of CNF clusters throughout the cement paste impacted the number of mechanically distinct peaks identified from statistical Gaussian fitting with the presence of a low stiffness, porous assemblage of phases that were not found in the reference cement paste and was attributed primarily to the interfacial zone surrounding the CNF clusters. The results furthermore revealed that the addition of CNFs favored higher stiffness C–S–H phases (indentation modulus of greater than 40 GPa) at the expense of the lower stiffness C–S–H (indentation modulus less than 30 GPa) and led to indentation modulus values for C–S–H that exceeded that of the high stiffness C–S–H. The indentation modulus of the cluster interfacial zone (a porous assemblage of CH and C–S–H phases), measured by nanoindentation, was in the range from 15 to 25 GPa, and the elastic modulus of the CNF clusters, estimated from the microscale–macroscale analytical homogenization method, ranged from 18 to 21 GPa. It was found that the CNF clusters acted as compliant inclusions relative to the matrix and that the CNF clusters and interfacial zone surrounding the clusters affected the macroscale elastic modulus of the cement paste. The nanoindentation-based microscale–macroscale upscaling methodology presented in this paper provided promising results to study the influence of randomly distributed inclusions at various length scales on the overall macroscale response of the composite.

## Figures and Tables

**Table 1:** Mean values of the indentation modulus and hardness and percent of indents for each mechanically distinct peak identified for the reference cement paste and the cement paste with CNFs.

		Indentation Modulus		Hardness	
		µ ± σ (GPa) ^1^	% of Indents	µ ± σ (GPa) ^1^	% of Indents
**Reference Cement Paste**	Peak A	28.7 ± 7.6 ^2^	77.5	1.2 ± 0.41	69.1
	Peak B	49.5 ± 6.8 ^2^	11.1	2.2 ± 0.41	18.1
	Peak C	64.6 ± 13.2 ^2^	6.5	3.7 ± 1.2	8.5
	Peak D	135.0 ± 52.0 ^2^	4.8	10.6 ± 3.6	4.3
**Cement Paste with CNFs**	Additional Peak	20.0 ± 4.2	10.2	0.4 ± 0.2	6.3
	Peak A	33.4 ± 4.8	63.3	1.2 ± 0.3	54.7
	Peak B	44.0 ± 5.0	16.3	1.7 ± 0.3	28.2
	Peak C	58.2 ± 5.8	4.0	3.1 ± 0.5	4.6
	Peak D	110.0 ± 15.0	6.3	6.2 ± 2.0	6.3

^1^ µ = mean; σ = standard deviation. ^2^ Data reported in [58].

**Table 2:** Classification of mechanically distinct phases (Additional Peak and Peaks A–D, this study) with their corresponding chemical phases as determined from indentation modulus and hardness measurements of cement phases reported in the literature from nanoindentation.

			Indentation Modulus (GPa)	Hardness (GPa)	References
**Additional Peak**	**Peak A**	Calcium silicate hydrate, C–S–H			
		Low stiffness	16.6 ± 4.7 ^3^	0.7 ± 0.1	Lee et al. [97]
			18.1 ± 4.0		Jennings et al. [91]
			18.2 ± 4.2	0.5 ± 0.1	Constantinides et al. [71]
			19.7 ± 2.5	0.6 ± 0.03	Sorelli et al. [52]
			20.0 ± 2.0	0.8 ± 0.2	Acker [98,99]
			21.7 ± 2.2		Constantinides et al. [53]
			22.9 ± 0.76	0.9 ± 0.1	Mondal et al. [60]
			23.4 ± 3.4	0.7 ± 0.2	Zhu et al. [92]
			23.7 ± 5.9	0.7 ± 0.2	Vandamme et al. [100]
			25.2 ± 2.8 ^1^	0.8 ± 0.3 ^1^	Hu et al. [101]
			30.1 ± 2.3 ^3^		Sebastiani et al. [93]
		High stiffness	29.1 ± 4.0		Constantinides et al. [71]
**Peak B**			29.4 ± 2.4	0.8 ± 0.2	Constantinides et al. [53]
			29.8 ± 4.0		Lee et al. [97]
			31.0 ± 4.0	1.4 ± 0.2	Jennings et al. [91]
			31.0 ± 4.0	0.9 ± 0.3	Acker [98,99]
			31.2 ± 2.5	1.2 ± 0.1	Mondal et al. [60]
			31.4 ± 2.1	1.3 ± 0.2	Zhu et al. [92]
			31.6 ± 2.9 ^2^	1.1 ± 0.2 ^2^	Hu et al. [101]
			34.2 ± 5.0	1.4 ± 0.4	Sorelli et al. [52]
			36.1 ± 3.4	1.0 ± 0.2	Vandamme et al. [100]
			39.3 ± 2.8 ^4^		Sebastiani et al. [93]
		Ultra-high stiffness	41.5 ± 1.8	1.4 ± 0.3	Mondal et al. [60]
			42.8 ± 2.3	1.4 ± 0.2	Vandamme et al. [82]
			47.2 ± 6.0	1.6 ± 0.3	Vandamme et al. [100]
		Calcium hydroxide, CH	36.0 ± 3.0	1.4 ± 0.5	Acker [98,99]
	**Peak C**		38.0 ± 5.0		Constantinides et al. [53]
			40.3 ± 4.2	1.3 ± 0.2	Constantinides et al. [71]
			48.7 ± 10.5	2.4 ± 1.2	Lee et al. [97]
		Unhydrated cement particles			
		Alite	125 ± 7	9.2 ± 0.5	Velez et al. [89]
**Peak D**		Tetracalcium aluminoferrite, 4CaO·Al_2_O_3_·Fe_2_O_3_ (C_4_AF)	125 ± 25	9.5 ± 1.4	Velez et al. [89]
			125 ± 25	9.5 ± 3.0	Acker [98,99]
		Belite	127 ± 10	8.8 ± 1.0	Velez et al. [89]
		Dicalcium silicate, 2CaO·SiO_2_ (C_2_S)	130 ± 20	8.0 ± 1.0	Velez et al. [89]
			130 ± 20	8.0 ± 2.0	Acker [98,99]
		Tricalcium silicate, 3CaO·SiO_2_ (C_3_S)	135 ± 7	8.7 ± 0.5	Velez et al. [89]
			135 ± 7	8.7 ± 1.0	Acker [98,99]
		Tricalcium aluminate, 3CaO·Al_2_O_3_ (C_3_A)	145 ± 10	10.8 ± 0.7	Velez et al. [89]
			145 ± 10	10.8 ± 1.5	Acker [98,99]

^1^ Outer product; ^2^ Inner product; ^3^ Low density; ^4^ High density.

**Table 3:** Output from the Gaussian fitting algorithm for the 0–50 GPa indentation modulus range of the phases typically associated with C–S–H phases for the reference cement paste and cement paste with CNFs.

	Reference Cement Paste		Cement Paste w/CNFs		Comparison to Literature	
	Indentation Modulus µ ± σ (GPa)	% of indents	Indentation Modulus µ ± σ (GPa)	% of indents	Indentation Modulus (GPa)	
**Phase 1 ^1^**	22.2 ± 3.5	25.9%	28.3 ± 2.0	18.9%	Low stiffness C–S–H	18.0–24.0 [52,60,91,92,100]
**Phase 2 ^2^**	31.2 ± 5.2	65.5%	34.1 ± 2.9	56.6%	High stiffness C–S–H	31.0–36.5 [52,60,91,92,100]
**Phase 3 ^3^**	45.7 ± 3.5	8.6%	44.3 ± 3.5	10.5%	Ultra-high stiffness C–S–H	41.0–47.5 [60,100]
**Additional Phase**	-	-	39.8 ± 1.8	14.0%	Intermediate C–S–H phase (between high and ultra-high)	(this paper)

^1^ Phase 1 likely corresponded to low stiffness C–S–H. ^2^ Phase 2 likely corresponded to high stiffness C–S–H. ^3^ Phase 3 likely corresponded to ultra-high stiffness C–S–H.

**Table 4:** Input parameters from grid nanoindentation data plus a phase for the CNF clusters and calculated overall homogenized elastic modulus of the cement paste with CNFs from the microscale–macroscale analytical homogenization method.

		Indentation Modulus (GPa)	Volume Fraction (%)	Adjusted Volume Fraction (%)	Poisson’s Ratio
Model Parameters	Cluster Phase		-	5.0	0.1–0.4
	Cluster Interfacial Zone	20.0	11.0	9.7	0.3
	Phase A	33.4	66.6	58.7	0.3
	Phase B	44.0	16.5	14.5	0.3
	Phase C	58.2	4.2	3.7	0.3
	Phase D	110.0	1.8	1.6	0.3
	Porosity ^1^	-	-	6.8	-
Estimated Model Parameter	Cluster Phase	18.0–21.0			
	**Macroscale Modulus Values**				
Homogenized Elastic Modulus (GPa)	With cluster phase				29.5
	Without cluster phase				31.7
Measured Elastic Modulus (GPa)	By pulse velocity				29.5 ± 0.5

^1^ Porosity = Corrected SEM Porosity using a correction factor of 0.6 to account for the fraction of porosity outside the SEM domain as detailed in [58].

## Data Availability

The data presented in this study are available on request from the corresponding author.

## Supplementary Materials

The following are available online at https://www.mdpi.com/article/10.3390/nano12020223/s1, Figure S1: (**a**) A representative load–displacement curve, (**b**) load–displacement curve that exhibits stiffening, and (**c**) load–displacement curve that exhibits jumps in displacement due to indenting on the entangled mass of CNFs within a cluster or improper tip contact at the boundary between two phases that present a large contrast in their mechanical behavior, Figure S2: Histogram of the normalized raw data for the (**a**,**b**) indentation modulus and (**c**,**d**) hardness of the reference cement paste and cement paste with CNFs, respectively, Figure S3: Comparison of the empirical cumulative density function (CDF) and theoretical CDF for the (**a**,**b**) indentation modulus and (**c**,**d**) hardness of the reference cement paste and cement paste with CNFs, respectively, Figure S4: Comparison of the empirical cumulative density function (CDF) and theoretical CDF for the 0 to 50 GPa range of the indentation modulus typically associated with C–S–H phases for the (**a**) reference cement paste and (**b**) cement paste with CNFs.
