# Supplementary material for: Effect of Carbon Nanofiber Clustering on the Micromechanical Properties of a Cement Paste
Source: Nanomaterials (Basel). 2022 Jan 10;12(2):223. doi: 10.3390/nano12020223 (PMC8778676; doi:10.3390/nano12020223)
Supplement: Supplementary file 1 [file nanomaterials-12-00223-s001.zip › nanomaterials-1491965-supplementary revised.pdf]

nanomaterials

MDPI

Supplementary Information

Effect of Carbon Nanofiber Clustering on the Micromechanical Properties of a Cement Paste

Lesa Brown [1], Catherine S. Stephens [2], Paul G. Allison [3] and Florence Sanchez [1,*]

[1] Department of Civil and Environmental Engineering, Vanderbilt University, Nashville, TN 37212, USA; lesa.brown@vanderbilt.edu
[2] International Research Office, U.S. Army Engineer Research and Development Center, Ruislip HA4 7HB, UK; Catherine.s.stephens.civ@army.mil
[3] Department of Mechanical Engineering, The University of Alabama, Tuscaloosa, AL 35401, USA; pallison@eng.ua.edu
* Correspondence: florence.sanchez@vanderbilt.edu; Tel.: +1-615–322–5135

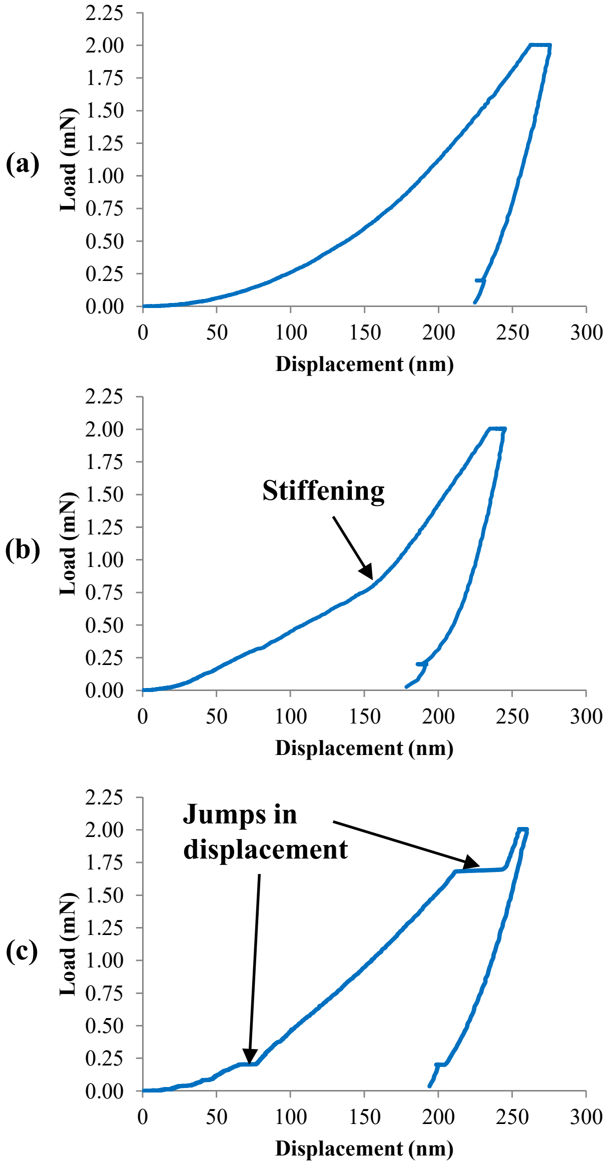

Figure S1. (**a**) A representative load-displacement curve, (**b**) load-displacement curve that exhibits stiffening, and (**c**) load-displacement curve that exhibits jumps in displacement due to indenting on

the entangled mass of CNFs within a cluster or improper tip contact at the boundary between two phases that present a large contrast in their mechanical behavior.

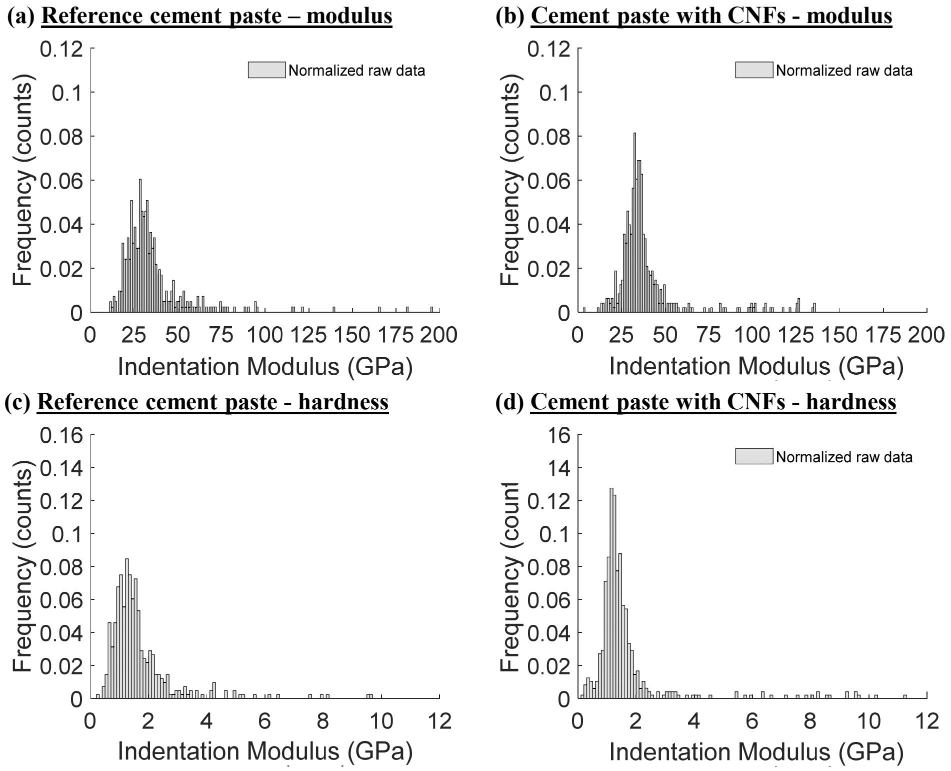

Figure S2. Histogram of the normalized raw data for the (**a**,**b**) indentation modulus and (**c**,**d**) hardness of the reference cement paste and cement paste with CNFs, respectively.

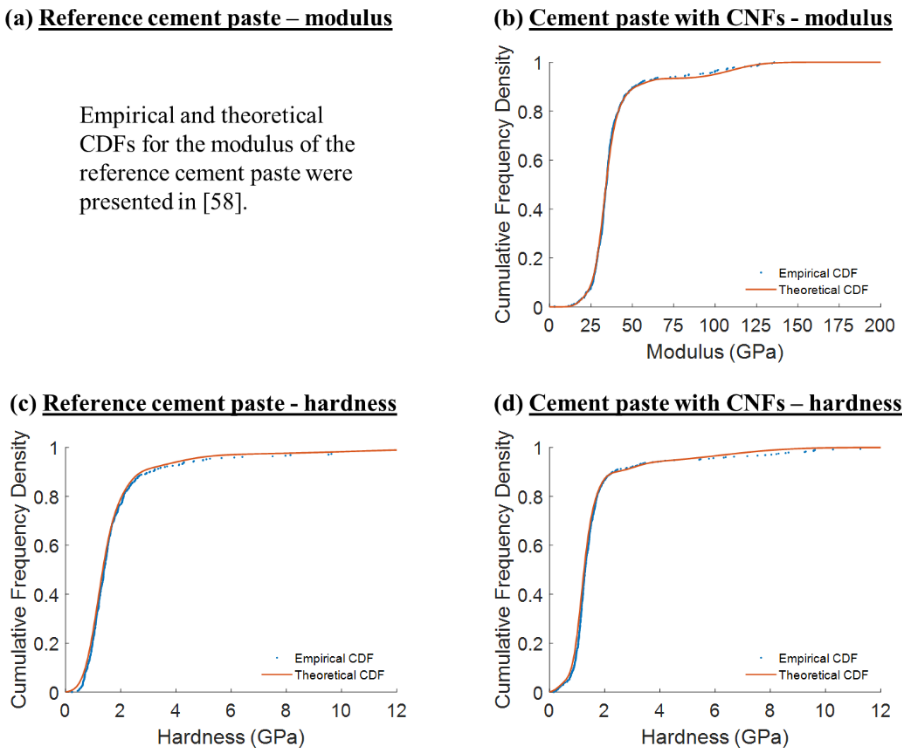

Figure S3. Comparison of the empirical cumulative density function (CDF) and theoretical CDF for the indentation modulus (**a**,**b**) and hardness (**c**,**d**) of the reference cement paste and cement paste with CNFs, respectively.

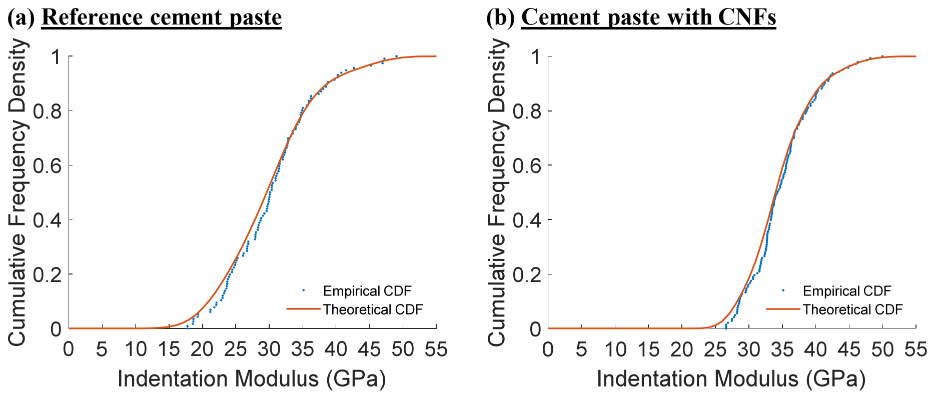

Figure S4. Comparison of the empirical cumulative density function (CDF) and theoretical CDF for the 0 to 50 GPa range of the indentation modulus typically associated with C-S-H phases for the (**a**) reference cement paste and (**b**) cement paste with CNFs.
